# Tiam1-Rac1 Axis Promotes Activation of p38 MAP Kinase in the Development of Diabetic Retinopathy: Evidence for a Requisite Role for Protein Palmitoylation

**DOI:** 10.1159/000374065

**Published:** 2015-04-30

**Authors:** Rajakrishnan Veluthakal, Binit Kumar, Ghulam Mohammad, Anjaneyulu Kowluru, Renu A. Kowluru

**Affiliations:** a Ophthalmology, Wayne State University; b Pharmaceutical Sciences, Wayne State University; c β-Cell Biochemistry Laboratory, John D. Dingell VA Medical Center, Detroit, MI, USA

**Keywords:** NSC23766, 2-bromopalmitate, p38 MAP kinase, Nox2, Rac1, Tiam1 and Diabetic, Retinopathy

## Abstract

**Background/Aims:**

Evidence in multiple tissues, including retina, suggests generation of reactive oxygen species (ROS) and the ensuing oxidative stress as triggers for mitochondrial defects and cell apoptosis. We recently reported novel roles for Tiam1-Rac1-Nox2 axis in retinal mitochondrial dysfunction and cell death leading to the development of diabetic retinopathy. Herein, we tested the hypothesis that activation of p38 MAP kinase, a stress kinase, represents the downstream signaling event to Rac1-Nox2 activation in diabetes-induced metabolic stress leading to capillary cell apoptosis.

**Methods:**

Activation of p38 MAP kinase was quantified by Western blotting in retinal endothelial cells incubated with high glucose (20 mM) for up to 96 hours, a duration where mitochondrial dysfunction and capillary cell apoptosis can be observed. NSC23766 and 2-bromopalmitate (2-BP) were used to assess the roles of Tiam1-Rac1 and palmitoylation pathways, respectively.

**Results:**

Activation of p38 MAP kinase was observed as early as 3 hours after high glucose exposure, and continued until 96 hours. Consistent with this, p38 MAP kinase activation was significantly higher in the retina from diabetic mice compared to age-matched normal mice. NSC23766 markedly attenuated hyperglycemia-induced activation of p38 MAP kinase. Lastly, 2-BP inhibited glucose-induced Rac1, Nox2 and p38 MAP kinase activation in endothelial cells.

**Conclusions:**

Tiam1-Rac1-mediated activation of Nox2 and p38 MAP kinase constitutes early signaling events leading to mitochondrial dysfunction and the development of diabetic retinopathy. Our findings also provide the first evidence to implicate novel roles for protein palmitoylation in this signaling cascade.

## Introduction

Diabetic retinopathy is a multi-factorial disease, and the mechanisms underlying the pathogenesis of this slow-progressing disease remain only partially understood. Our work has suggested that in the pathogenesis of diabetic retinopathy, an increase in oxidative stress in the retina and its capillary cells is observed before mitochondria become dysfunctional and capillary cell apoptosis is accelerated [1–5]. We have recently reported novel regulatory roles of NADPH oxidases (Noxs), specifically the phagocyte-like Nox2, in glucose-induced generation of reactive oxygen species (ROS) in the retina and its capillary cells, our study has demonstrated that diabetes activates Nox2 and increases ROS levels before damaging mitochondria and its DNA (mtDNA). Furthermore, NSC23766, a known inhibitor of Rac1 (via inhibiting Rac1 interaction with its guanine nucleotide exchange factor, Tiam1), which is a member of Nox2 holoenzyme, markedly attenuates Rac1 activation, mitochondrial damage and cell apoptosis in retinal endothelial cells, thus affirming regulatory roles for Nox2 as a source for ROS generation to trigger mitochondrial damage and accelerated cell death [6].

**Fig. 1:**
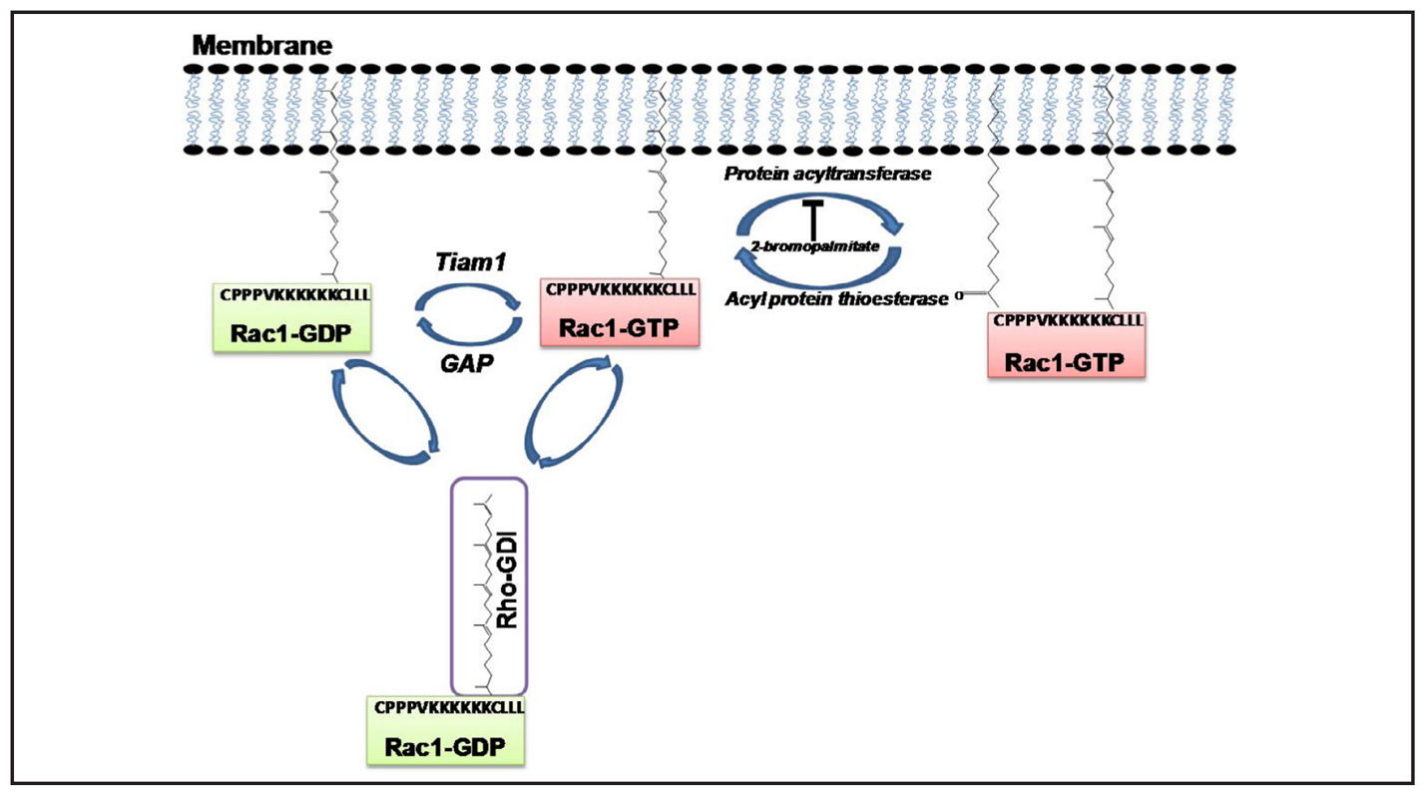
A schematic representation of post-translational modification of Rac1. The majority of small G-proteins (e.g., members of Rho subfamily, Rac1) undergo a series of post-translational modifications at their C-termini, including prenylation and carboxylmethylation [8–11, 46]. In addition, certain G-proteins (Rac1) have also been shown to undergo palmitoylation, catalyzed by protein acyltransferase, at a cysteine residue, which is upstream to the prenylated cysteine. Palmitoylation provides a “firm” anchoring for the modified protein into the cell membrane for optimal interaction with its respective effector proteins [8–11, 46]. Depalmitoylation of these proteins is catalyzed by acyl protein thioesterase. Recent evidence implicates that palmitoylation also promotes Rac1 activation (GTP-bound conformation). Also shown here is activation-deactivation cycle for Rac1. Exchange of GDP for GTP is mediated by Tiam1, a known, guanine nucleotide exchange factor for Rac1. In the current study, we examined putative roles of Tiam1-Rac1 axis (NSC23766) and protein palmitoylation (2-bromopalmitate; 2-BP) in glucose-induced p38 MAP kinase phosphorylation and activation (see text for additional details). Abbreviations used are: Rac1: Ras-related C3 botulinum toxin substrate 1; Rac1-GDP: Rac1 bound to guanosine diphosphate [inactive]; Rac1-GTP: Rac1 bound to guanosine triphosphate; GAP: GTPase activating protein; GDI: guanosine diphosphate dissociation inhibitor; and Tiam1: T-cell lymphoma invasion and metastasis 1.

A majority of small molecular mass G-proteins, such as Rac1, undergo a series of post-translational modifications at their C-terminal cysteine residues, including prenylation and methylation, which render the modified proteins more hydrophobic, thus promoting their translocation to the membranous compartment for optimal regulation of effector proteins (Fig. 1) [7–10]. In addition, the activation-deactivation cycle is regulated by a variety of regulatory proteins and factors, including GTP-GDP exchange factors (GEFs; Tiam1 for Rac1), GDP-dissociation inhibitor (Rho-GDI), and GTPase-activating proteins (GAP) [11–13]. Earlier studies from our laboratory have demonstrated that small G-proteins belonging to the Ras superfamily (H-Ras) undergo additional post-translational modifications at cysteine residues upstream to the prenylated cysteine [14–16]. These include *S*-acylation (palmitoylation), in which protein acyltransferases facilitate incorporation of palmitic acid into cysteine residues *via* thioester linkages (Fig. 1). Using selective inhibitors (cerulenin and 2-Bromopalmitic acid; 2-BP), we have demonstrated that palmitoylation promotes association of H-Ras into organized lipid rafts (caveolin-1 enriched fraction) in the islet β-cell. More recent studies by Navarro-Lerida have also demonstrated that Rac1 undergoes palmitoylation at cysteine-178, which, in turn, promotes its translocation to the ordered membrane regions, and the non-palmitoylated Rac1 exhibits decreased GTP-loading (activation) and membrane association [17].

Diabetes induces stress kinase (p38 MAP kinase) activation to induce metabolic dysfunction in multiple cell types, including the retinal endothelial and capillary epithelial cells [18–23]. Along these lines, we recently proposed that accelerated Tiam1-Rac1-Nox2 signaling axis could also contribute to the stress kinase activation in these cells [6, 24]. The current study, therefore, is aimed at assessing the roles of p38 MAP kinase as downstream signaling events to glucose-induced Rac1-Nox2 activation. We addressed this by asking if pharmacological inhibition of Tiam1-Rac1 signaling (NSC23766; [*N*^6^-[2-[[4-(Diethylamino)-1-methylbutyl]amino]-6-methyl-4-pyrimidinyl]-2-methyl-4,6-quinolinediamine trihydrochloride]) and palmitoylation (2-BP; Fig. 1) [7–9] on glucose-induced p38 MAP kinase activation. Our findings suggest that Rac1-mediated activation of Nox2 and p38 MAP kinase constitute early signaling events leading to mitochondrial dysfunction and the development of diabetic retinopathy, and provide the first evidence to implicate novel roles for protein palmitoylation in this signaling cascade in the development of diabetic retinopathy.

## Materials and Methods

### Retinal endothelial cells

Endothelial cells, isolated from bovine retina, were incubated in Dulbecco’s Modified Eagle Medium consisting of 2% heat inactivated fetal bovine serum, 10% Nu-serum, 50 μg/ml heparin, 1 μg/ml endothelial growth factor and antibiotic/anti-mycotic for 3–96 hours containing 5 or 20mM glucose [or 20 mM mannitol as an osmotic control], in the absence or presence of 20 μM of NSC23766 (Calbiochem-EMD Millipore, Billerica, MA) or 100 μM of 2-BP (Sigma-Aldrich Chemicals, St. Louis, MO), as routinely performed in our laboratory [6, 25].

### Experimental Animals

Male C57BL/6J mice (~20 g, 6–7 weeks old) obtained from Jackson Laboratory (Bar Harbor, ME), were injected streptozotocin (STZ, 55 mg/kg) for 5 consecutive days [1, 25]. Those mice presenting with a blood glucose concentration of 14 mmol/l or higher, 1 day after the last injection of STZ, were considered to be diabetic. A group of diabetic mice was administered NSC23766 (2.5 mg kg^−1^ day^−1^, i.p; Sigma-Aldrich, St. Louis, MO) soon after establishment of diabetes. The mice were killed 2 weeks after initiation of NSC23766 treatment by carbon dioxide asphyxiation and one eye was used to isolate the retina and frozen immediately in liquid nitrogen for biochemical analysis, and the other eye was incubated in 10% formaldehyde for 30 min, washed with PBS, fixed in optimal cutting temperature compound (OCT), and frozen in liquid nitrogen for sectioning and immunostaining [26]. Age-matched normal mice served as controls. The treatment of the animals conformed to the Association for Research in Vision and Ophthalmology Resolution on the Use of Animals in Research, and institutional guidelines.

### p38 MAP kinase activation

Activation of p38 MAP kinase was determined by Western blotting in retinal endothelial cells incubated under various experimental conditions using an antibody raised against a short amino acid sequence containing dually phosphorylated Thr 180/Tyr 182 of p38α (Santa Cruz Biotechnology, Santa Cruz, CA). The same blots were stripped and re-probed with total p38 MAP kinase (Santa Cruz Biotechnology, Santa Cruz, CA). Relative intensities of the bands were quantified by densitometry.

### Nox2 activity

Nox2 activity was measured in the cell homogenate by luminescence assay using 20 μM lucigenin as electron acceptor and 100 μM NADPH [6, 27, 28].

### Rac1 activation assay

Rac1 activation was quantified using a G-LISA assay kit (Cytoskeleton Inc., Denver, CO) as described in [13, 29].

### ROS generation assay

Total ROS levels were measured fluorometrically by incubating 5 μg of protein with 2 μmol/l of 2′,7′ dichlorofluorescein diacetate, DCHFDA (Sigma-Aldrich, St. Louis, MO) for 10 minutes. The fluorescence was measured at 485 nm and 530 nm as excitation and emission wavelengths, respectively [6].

### Mitochondrial DNA damage

Extended length PCR was performed by amplifying long (13.4kb) and short (210bp) regions of mtDNA. Total DNA was isolated using DNeasy blood and tissue kit (Qiagen, Valencia, CA), and the ratio between the intensity of the long to short fragment of PCR amplicons was calculated [5, 6, 30].

### Quantification of cell apoptosis

Apoptosis was performed using an ELISA kit from Roche Diagnostics (Indianapolis, IN) as routinely used in our laboratory. Briefly, mono- and oligonucleosomes generated from the apoptotic cells were quantified using monoclonal antibodies directed against DNA and histones respectively. Using 2,2′-Azino-di-[3-ethylbenzthiazoline sulfonate] diammonium salt, the absorbance generated was measured at 405 nm [6, 25].

### Immunostaining of retinal cryosections

Mouse retina cryosections (10 μm) were fixed in 4% paraformaldehyde and blocked with 10% normal goat serum. The sections were rinsed with PBS, permeabilized with 0.1% Triton X-100, and incubated over night with anti-p38 antibody (rabbit polyclonal, ab7952, Abcam; Cambridge, MA). The slides were rinsed with PBS, and incubated with the anti-rabbit-FITC conjugated secondary antibody (NL006; R&D systems; Minneapolis, MN) for 1 hour [26]. After rinsing the slides with PBS, the sections were mounted with DAPI-containing mounting media (Vector Laboratories; Burlingame, CA), and imaged with an Olympus BX-UCB fluorescent microscope (20X magnification). The fluorescence intensity was quantified by using ImageJ software (version 1.44; NIH). Cryosections processed under similar conditions, except being incubated with the primary antibody, served as controls.

### Statistical analysis of experimental data

Statistical analysis was carried out using Sigma Stat software. Data are analyzed by multiple comparisons and expressed as mean ± SD. A *p*-value of < 0.05 was considered statistically significant.

## Results

### Inhibition of Tiam1-Rac1 signaling axis ameliorates p38 MAP kinase activation in diabetic mice

**Fig. 2:**
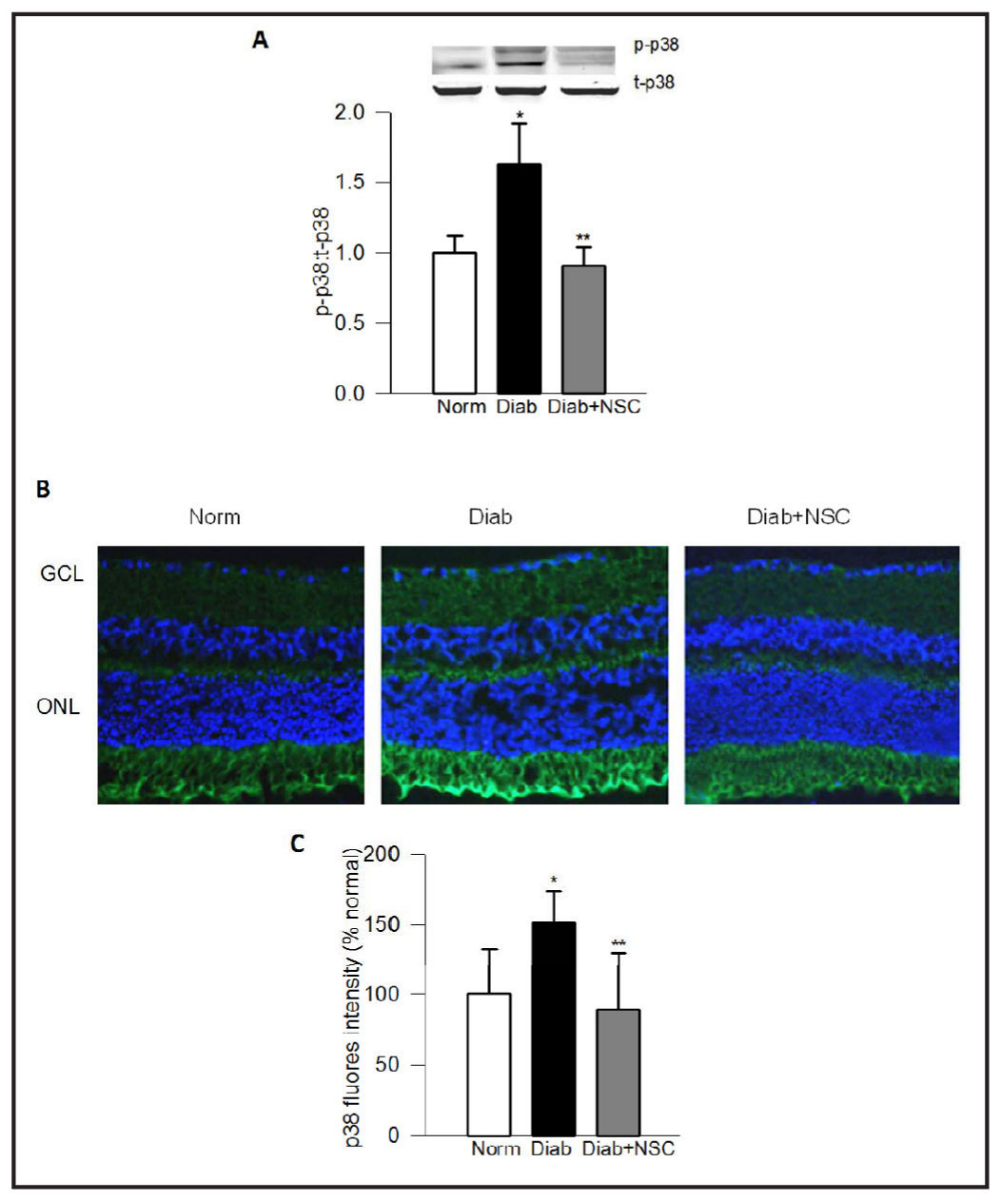
NSC23766 administration protects diabetes-induced p38 MAP kinase in mouse retina. Panel A. Retinal proteins from normal, diabetic and diabetic mice treated with NSC23766, a specific inhibitor of Tiam1-Rac1 signaling pathway [6], were separated by SDS-PAGE and the relative abundance of phosphorylated (p-p38) and total (t-p38) p38 MAP kinase was determined by Western blotting. Fold increase in the ratios of p-p38 to t-p38 are shown here. Panel B. Immunostaining of p38 MAP kinase was performed in the 10μm thick retinal cryosections using anti-p38 antibody (green), and DAPI (blue) was used to stain the nuclei. The sections were imaged at 20X magnification using Olympus BX50 fluorescent microscope. The images presented here are representative of 3 or more mice in each group. Panel C represents the fluorescence intensity, quantified by using ImageJ software. Norm=Normal, Diab=Diabetes and Diab+NSC= diabetic mice receiving NSC23766. ONL and GCL= Outer nuclear layer and ganglion cell layer respectively. The data are mean ± SEM from four animals in each group. *P < 0.001 *vs.* normal and **P < 0.001 *vs.* diabetic.

We have recently reported significant inhibitory effects of NSC23766, a known inhibitor of Tiam1-Rac1 signaling pathway, on Rac1 activation, Nox2 activity and ROS generation in endothelial cells and the retina of diabetic mice [6]. As a logical extension we further assessed the relevance of p38 MAP kinase activation by Tiam1-Rac1 signaling axis by quantifying p38 MAP kinase activation in retina from diabetic mice treated without NSC23766 [6]. Fig. 2 (Panel A) demonstrated ~50% increase in p-p38 MAP kinase in the retina from the diabetic mice, which was completely abrogated in diabetic mice receiving NSC23766, an inhibitor of Tiam1-Rac1 signaling pathway, soon after induction of diabetes. Furthermore, NSC23766 administration also decreased its diabetes-induced increased localization of p38 MAP kinase in the retinal vasculature (Panels B and C), as evidenced by the decreased intensity of the immunostaining.

### Tiam1-Rac1 signaling pathway mediates glucose-mediated activation of p38 MAP kinase in retinal endothelial cells

**Fig. 3:**
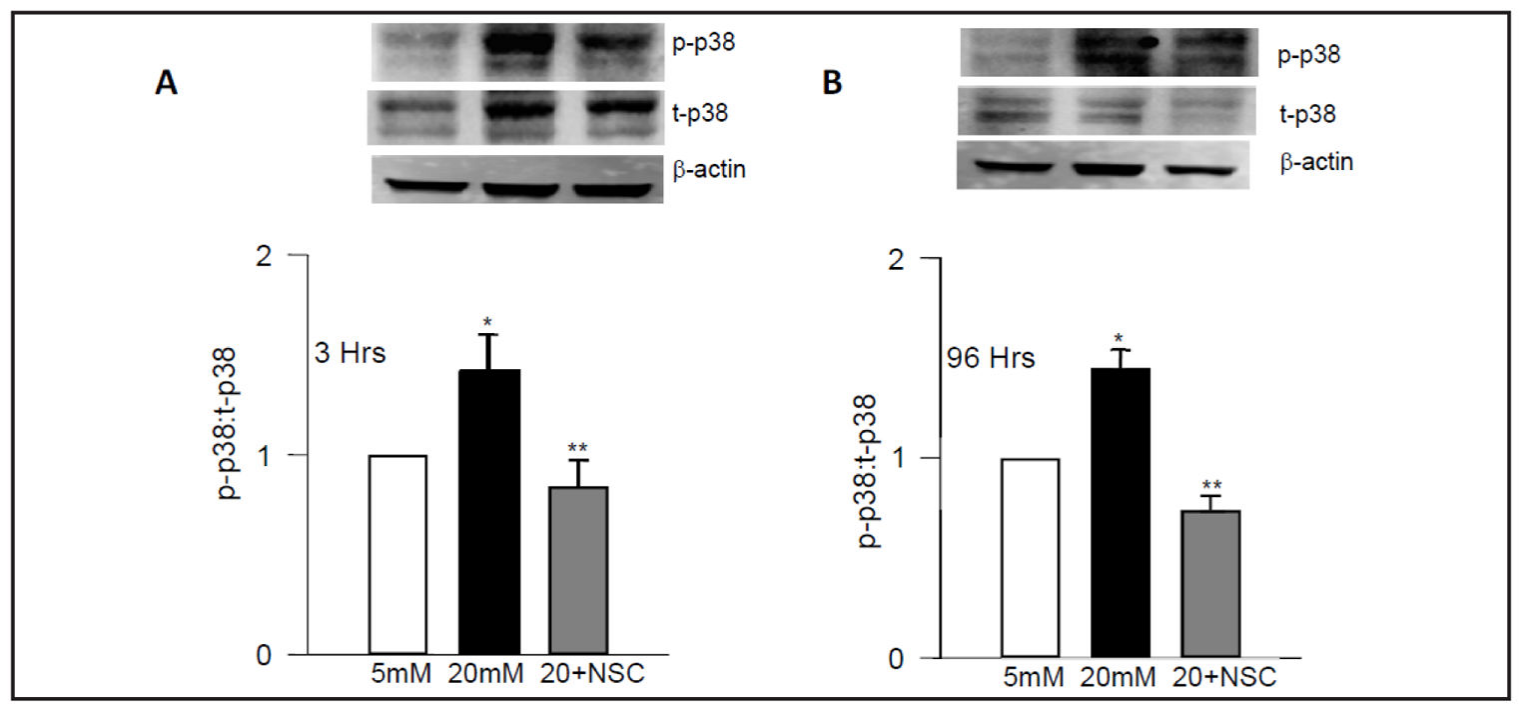
Tiam1-Rac1 signaling pathway mediates glucose-induced p38 MAP kinase. Retinal endothelial cells were incubated for 3 hours (Panel A) or 96 hours (Panel B) in 5 mM or 20 mM glucose in the absence or presence of NSC23766 (20 μM). Abundance of phosphorylated (p-p38) p38 and total (t-p38) p38 MAP kinase was determined in homogenates by Western blotting. A representative blot is shown here. Western blots for β-actins, as loading controls, are also provided in the Fig.. The accompanying histograms represent the ratios of p-p38 to t-p38 MAP kinase. Data are mean ± SD from four independent experiments. 5mM and 20mM=cells incubated in 5mM glucose or 20mM glucose respectively. *P < 0.001 *vs.* 5mM glucose and **P < 0.001 *vs.* 20mM glucose.

Within 3 hours of incubation of endothelial cells with high glucose, p38 MAP kinase was activated by ~50% (Fig. 3; Panel A), which remained at that level for up to 96 hours of incubation (Fig. 3; Panel B). NSC23766 abolished glucose-induced activation of p38 MAP kinase at both 3 and 96 hour time points. It is noteworthy that NSC23766 has been shown to inhibit glucose-induced Rac1 activation in endothelial cells under these conditions [6]. Together, these results suggest that p38 MAP kinase represents one of the down-stream signaling events mediated by Tiam1-Rac1 signaling pathway under the duress of hyperglycemic conditions.

### Inhibition of protein palmitoylation attenuates glucose-mediated activation of Nox2 and ROS generation

**Fig. 4:**
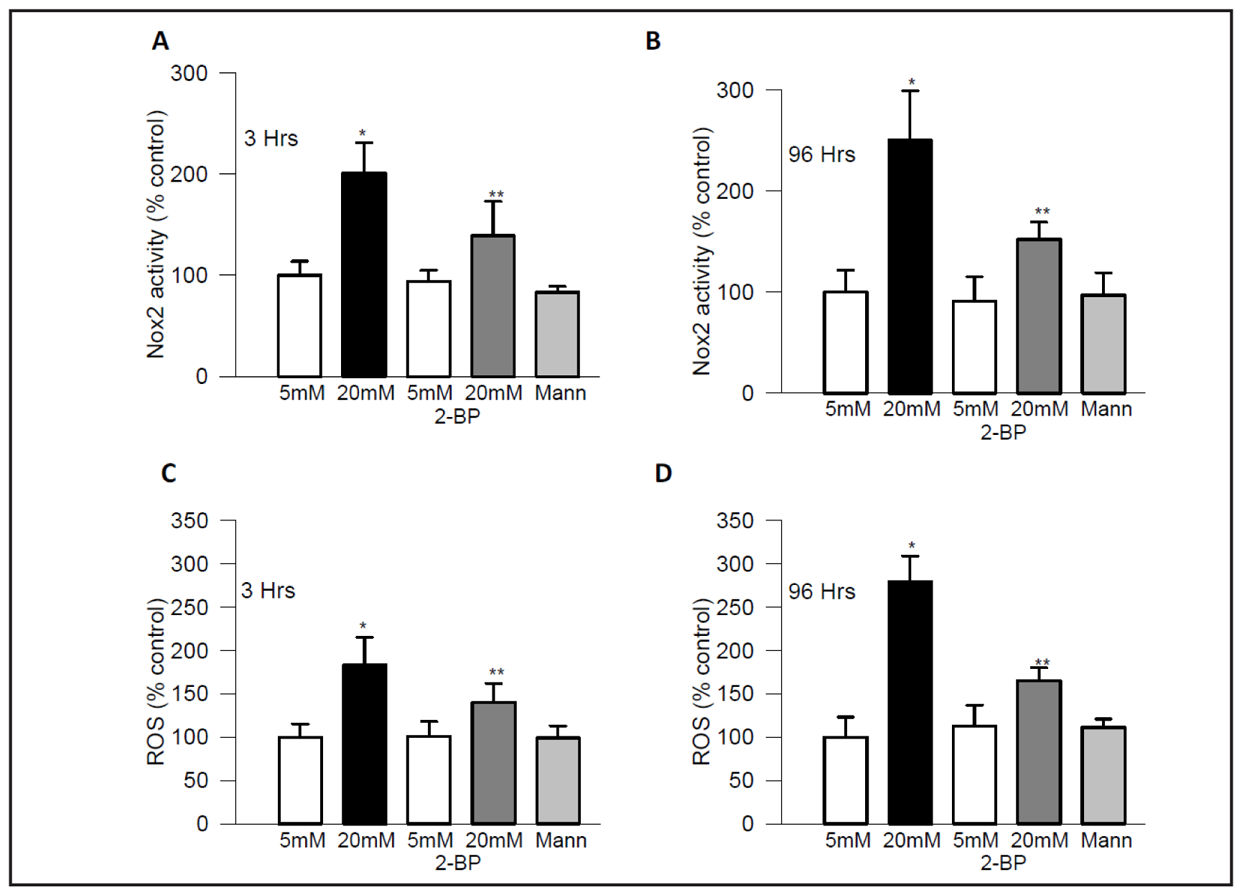
Protein palmitoylation is necessary for activation of Nox2 and ROS generation. Endothelial cells, incubated in normal or high glucose for 3 hours or 96 hours in the absence or presence of 2-bromopalmitate (2-BP), were analyzed for Nox2 activity (Panels A & B respectively), and for quantification of ROS levels using DCHFDA (Panels C & D respectively). 5mM and 20mM=cells incubated in 5mM glucose or 20mM glucose, respectively and Mann= cells incubated in mannitol [as an osmotic control]. *P < 0.001 *vs.* 5mM glucose and **P < 0.001 *vs*. 20mM glucose.

We next assessed if palmitoylation-derived activation of proteins (e.g., Rac1) regulates Nox2 activation in high glucose conditions, since Rac1 is an integral part of holoenzyme complex [31–34], and NSC23766 inhibits glucose-induced activation of Nox2. Consistent with our recent results [6, 31–34], incubation of endothelial cells with high glucose for 3–96 hours significantly increased Nox2 activity and ROS levels, and addition of 2-BP, a specific inhibitor of protein palmitoylation [28, 35, 36], in the incubation medium, abolished glucose-induced Nox2 activity and ROS levels (Fig. 4). 2-BP had no effect on Nox2 and ROS in the cells incubated in normal glucose. Taken together, these data suggest importance of a palmitoylation-dependent signaling step in glucose-induced Rac1 and Nox2 activation.

### Palmitoylation is requisite for glucose-induced Rac1 activation

**Fig. 5:**
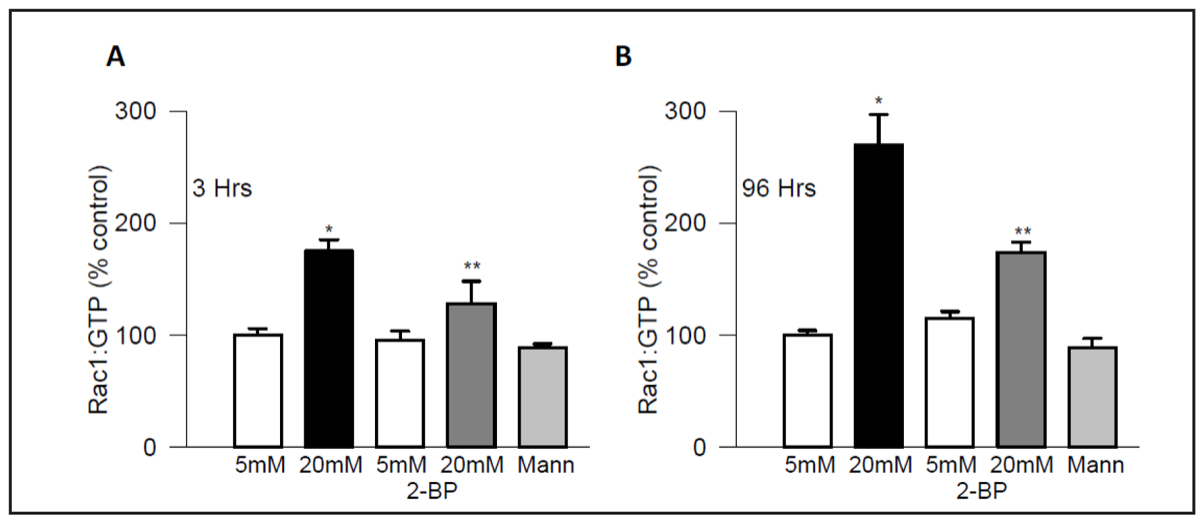
2-BP inhibits glucose-induced Rac1 activation in endothelial cells. Rac1 activation was quantified by G-LISA in the retinal endothelial cells incubated in 5mM or 20mM glucose for 3 hours (Panel A) or 96 hours (Panel B), in the absence or presence of 2-BP (100 μM). Data are expressed as percent control, and are mean ± SD from four independent experiments. 5mM and 20mM=cells incubated in 5mM glucose or 20mM glucose, respectively and Mann= cells incubated in mannitol [as an osmotic control]. *P < 0.001 *vs.* 5mM glucose and **P < 0.001 *vs.* 20mM glucose.

As stated above, the majority of small molecular mass G-proteins undergo post-translational modifications at their C-termini, including palmitoylation (Fig. 1). Such modifications render the modified G-proteins more hydrophobic, thereby enabling them to translocate to the membrane for the interaction/activation of their respective effector proteins [7–10]. Therefore, we asked if a palmitoylation-dependent signaling mechanism could be playing a role in Rac1 activation. Data shown in Fig. 5 indicate a significant increase in the activation of Rac1 (GTP-bound configuration) in cells incubated with high glucose for 3–96 hours, and addition of 2-BP completely inhibited glucose-induced Rac1 activation without significantly affecting Rac1 activation under basal conditions. These findings suggest that a palmitoylation-dependent signaling step controls Rac1 activation by high glucose in retinal endothelial cells.

### Inhibition of palmitoylation suppresses glucose-induced p38 MAP kinase

**Fig. 6:**
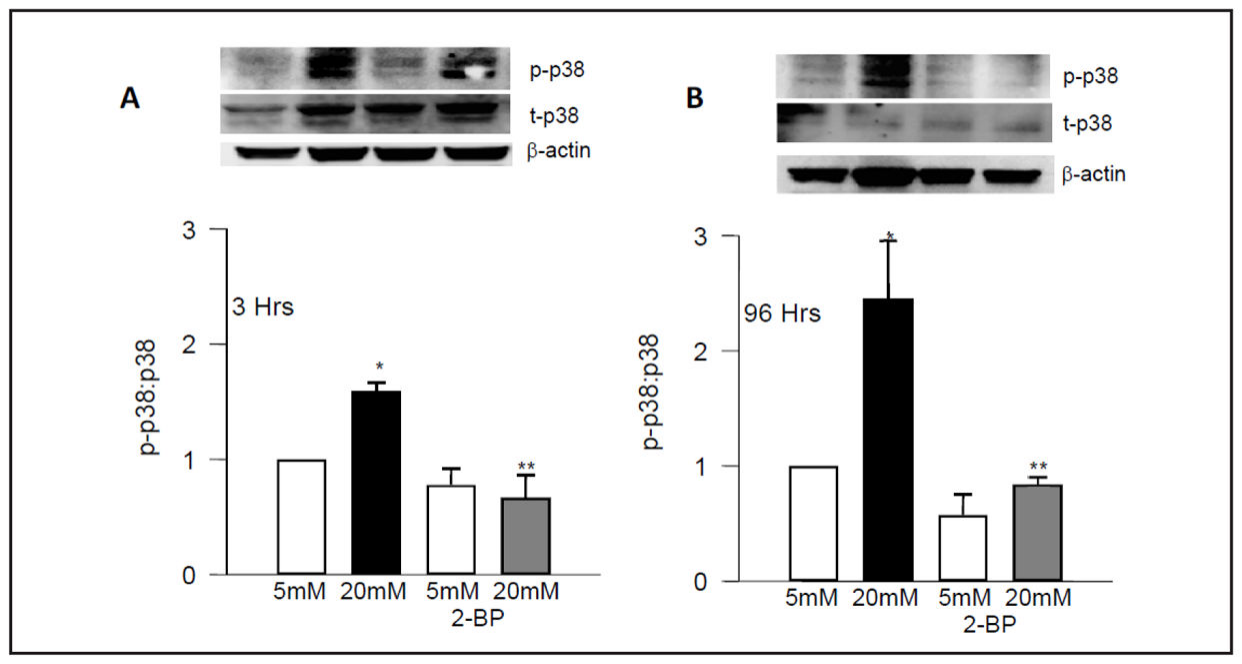
Protein palmitoylation is essential for activation of p38 MAP kinase. Endothelial cells were incubated in normal or high glucose for 3 hours (Panel A) or 96 hours (Panel B) in the absence or presence of 2-BP (100μM), and abundance of phosphorylated p38 (p-p38) and total (t-p38) p38 MAP kinase was determined by Western blotting. A representative blot is shown here. Western blots for β-actin, as loading controls, are also shown in the Fig.. Fold increase in the ratios of p-p38 to t-p38 are also shown in the accompanying histograms. Data are represented as mean ± SD from four independent experiments. 5mM and 20mM=cells incubated in 5mM glucose or 20mM glucose respectively *P < 0.001 *vs*. 5mM glucose and **P < 0.001 *vs.* 20mM glucose.

To determine the regulatory role of palmitoylation in p38 MAP kinase activation, effect of 2-BP on p38 MAP kinase was investigated. Data in Fig. 6 demonstrate a significant inhibition by 2-BP of glucose-induced p38 MAP kinase activation at both 3 hours (Panel A) and 96 hours (Panel B) of incubation.

### Inhibition of palmitoylation attenuates glucose-induced mtDNA damage and accelerated capillary cell apoptosis

**Fig. 7:**
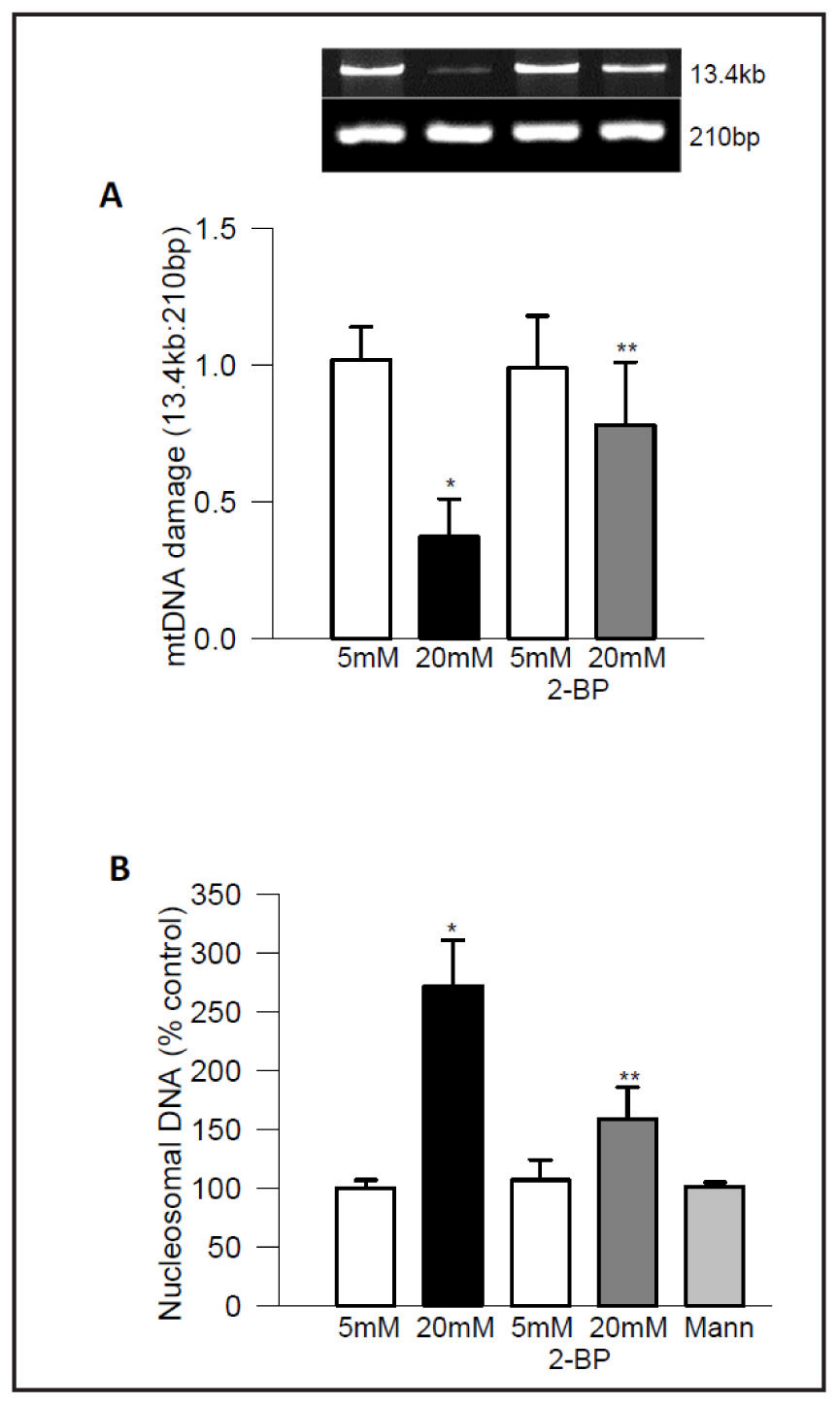
Inhibition of protein palmitoylation protects endothelial cells from glucose-induced mtDNA damage and accelerated apoptosis. Cells incubated in 5mM or 20mM glucose for 96 hours, in the presence or absence of 2-BP were analyzed for (Panel A) mtDNA damage by extended length PCR using primers for the long (13.4kb) and short (210bp) regions of mtDNA. The ratio between the long to short fragment of PCR amplicons was plotted in the histogram. Apoptosis (Panel B) was quantified by ELISA using the Cell Death Detection ELISA^PLUS^ kit, and the final absorbance generated by the incubation with 2,2′-Azino-di-[3-ethylbenzthiazoline sulfonate] was measured spectrophotometrically at 405 nm. Values are represented as mean ± SD of 3 or more experiments, with each measurement made in duplicate. 5mM and 20mM=cells incubated in 5mM glucose or 20mM glucose respectively and Mann= cell incubated in mannitol. **P < 0.001 *vs*. 5mM glucose and **P < 0.001 *vs.* 20mM glucose.

Since our recent results have shown that Nox2-mediated increase in ROS damages mitochondria and initiates the apoptotic process, the effect of 2-BP on mtDNA damage and cell apoptosis was investigated in cells exposed to high glucose for 96 hours. As shown in Fig. 7 (Panel A), addition of 2-BP ameliorated increase in mtDNA damage, as evidenced by amelioration of decrease in the ratio of 13.4kb and 210bp amplicons. In the same cell preparations, 2-BP decreased cell apoptosis (panel B). However, addition of 2-BP in 5mM glucose medium had no effect on either mtDNA damage or cell apoptosis.

## Discussion

In the pathogenesis of diabetic retinopathy, although increase in ROS is an early event, mitochondrial damage is not observed till the duration of diabetes is extended. Our recent studies have shown that the Tiam1-Rac1-Nox2 signaling module is activated in the initial stages of diabetes to increase intracellular ROS levels leading to mitochondrial damage and accelerated capillary cell apoptosis [6]. In the current study, we employed NSC23766, a specific inhibitor of Rac1, but not Cdc42 and RhoA [12], to further assess the roles of Tiam1, a known guanine nucleotide exchange factor for Rac1, in glucose-induced p38MAPK activation. We also demonstrated recently that NSC23766 selectively inhibits glucose-induced Rac1-Nox2-ROS signaling pathway in retinal endothelial cells [6]. As a logical extension of these findings, we undertook the current investigation to identify potential downstream signaling events to this pathway. Our findings suggest that: [i] inhibition of Tiam1-Rac1-Nox2 pathway (NSC23766) significantly attenuates hyperglycemia-induced p38 MAP kinase in the retina and its capillary cells and [ii] 2-BP, an irreversible inhibitor of S-acyltransferase, markedly attenuates Rac1-Nox2-p38 MAP kinase cascade. Collectively, these findings implicate novel roles for protein palmitoylation in the early signaling events which lead to the capillary cell apoptosis and the development of diabetic retinopathy.

Several recent studies have implicated accelerated p38 MAP kinase in metabolic dysregulation of retinal function in diabetes. Significant increases in p38 MAP kinase, ERK and inducible nitric oxide synthase (iNOS) activities have been reported in human retinal pigmented epithelial cells exposed to high glucose, and inhibition of p38 MAP kinase leads to significant reduction in iNOS gene expression and cell dysfunction. These studies have implicated damaging roles for MAP kinase in dysregulation of retinal pigment epithelial cells [37]. Using a specific inhibitor of p38 MAP kinase, Du *et al*. have shown an important role of MAP kinase in the development of early stages of diabetic retinopathy, and the mechanism appears to be *via* regulation of inflammation in the retina [38]. MAP kinase is also implicated in alterations in tight junction proteins, leukocyte adhesion, blood retinal barrier breakdown, and in the proNGF-mediated retinal neuronal apoptosis [39, 40], some of the early functional and structural abnormalities associated with diabetic retinopathy [41, 42]. We have shown that MAP kinase plays a significant role in activation of small molecular weight G-protein, H-Ras-mediated activation of matrix metalloproteinase-9 (MMP-9) in retinal capillary cells in diabetes; activated MMP-9 damages the mitochondria, allowing cytochrome-C to leak out and initiate the apoptosis process [25, 26, 43, 44], a phenomenon which precedes the development of histopathology characteristic of diabetic retinopathy [45]. Collectively, these studies implicate novel regulatory roles for p38 MAP kinase in the development of diabetic retinopathy.

Our current findings identify Tiam1-Rac1-Nox2 signaling axis as an upstream event in induction of p38 MAP kinase in retinal endothelial cells exposed to high glucose *in vitro*; our *in vivo* findings in retina from the diabetic mice confirmed these *in vitro* observations. We show that p38 MAP kinase is activated under the duress of high glucose within 3 hours of exposure and continues to be active till 96 hours of exposure. Furthermore, NSC23766, a known inhibitor of Tiam1-Rac1-Nox2 signaling pathway in the retina from diabetic mice [6], significantly attenuates p38 MAP kinase. Thus, these data establish a link between these two signaling pathways. More importantly, since the activation of p38 MAP kinase is demonstrable at a time point (3 hours), much earlier than the onset of mitochondrial dysfunction [4, 5], these data suggest that Nox2 signaling pathway-mediated increase in stress kinase activation triggers mitochondrial dysfunction and apoptosis of endothelial cells leading to diabetic retinopathy.

The current study also provide compelling evidence to implicate modulatory roles for protein palmitoylation in the onset of metabolic dysfunction induced by hyperglycemic conditions. Protein palmitoylation, catalyzed by S-acyltransferase, involves incorporation of palmitate into cysteine residues *via* a thioester linkages [7, 8]. Palmitoylation of small G-proteins takes place at cysteine residues upstream to prenylated cysteines [7, 8]. Using pharmacological and radiometric approaches, we have reported previously that H-Ras undergoes palmitoylation in the pancreatic islet β-cell, and that such a signaling step is necessary for iNOS gene expression and subsequent NO release under conditions of exposure to proinflammatory cytokines (IL-1β) [15, 46]. We have also demonstrated enhanced translocation of H-Ras to the membrane fraction, specifically into the caveolin-1 enriched lipid rafts. In this context, Navarro-Lerida and associates have reported palmitoylation of Rac1 at cysteine-178 residue and that such a modification is requisite for GTP-loading and membrane association of Rac1 [17]. 2-BP significantly attenuates GTP-loading and membrane-association of Rac1 suggesting requisite nature of palmitoylation in the regulation of Rac1 function [17]. Data accrued in our current investigations suggest that a palmitoylation-dependent signaling step is involved in high glucose-induced Rac1-Nox2-p38 MAP kinase signaling cascade. These conclusions are based on our findings that in pancreatic beta cells, 2-BP inhibits Rac1 activation, Nox2 activity and p38 MAP kinase phosphorylation and activation [28]. It should be noted that several cellular proteins, including Rac1 and Ras undergo palmitoylation. 2-BP is used in the current studies to determine if palmitoylation is one of the regulatory steps involved in high glucose-induced p38 MAP kinase activation. Our findings indicate that it inhibits glucose-induced Rac1 and p38 MAPK activation. Additional studies are needed, however, to conclusively demonstrate that palmitoylation of Rac1 mediates these signalling steps. These include quantification of high glucose-induced Rac1-Nox2-ROS-p38 MAPK signaling steps in endothelial cells expressing the palmitoylation resistant mutant of Rac1 (C178S Rac1) or in cells following siRNA-mediated knockdown of acyltransferase. Over expression of constitutively active (V12Rac1) and dominant negative (N17Rac1) might provide additional strength to our hypothesis. These studies, which are outside the framework of the current studies, are being pursued currently. Future studies, including those involving palmitoylation-resistant (C178S Rac1), constitutively active (V12Rac1) and dominant negative (N17Rac1) mutants of Rac1 and/or siRNA-mediated knock down of S-acyltransferase will conclusively validate our hypothesis that Rac1 palmitoylation and activation (GTP-loading) are necessary for its translocation to the organized membrane compartments leading to the assembly of Nox2 holoenzyme, ROS generation and metabolic dysregulation of retinal endothelial cells.

**Fig. 8:**
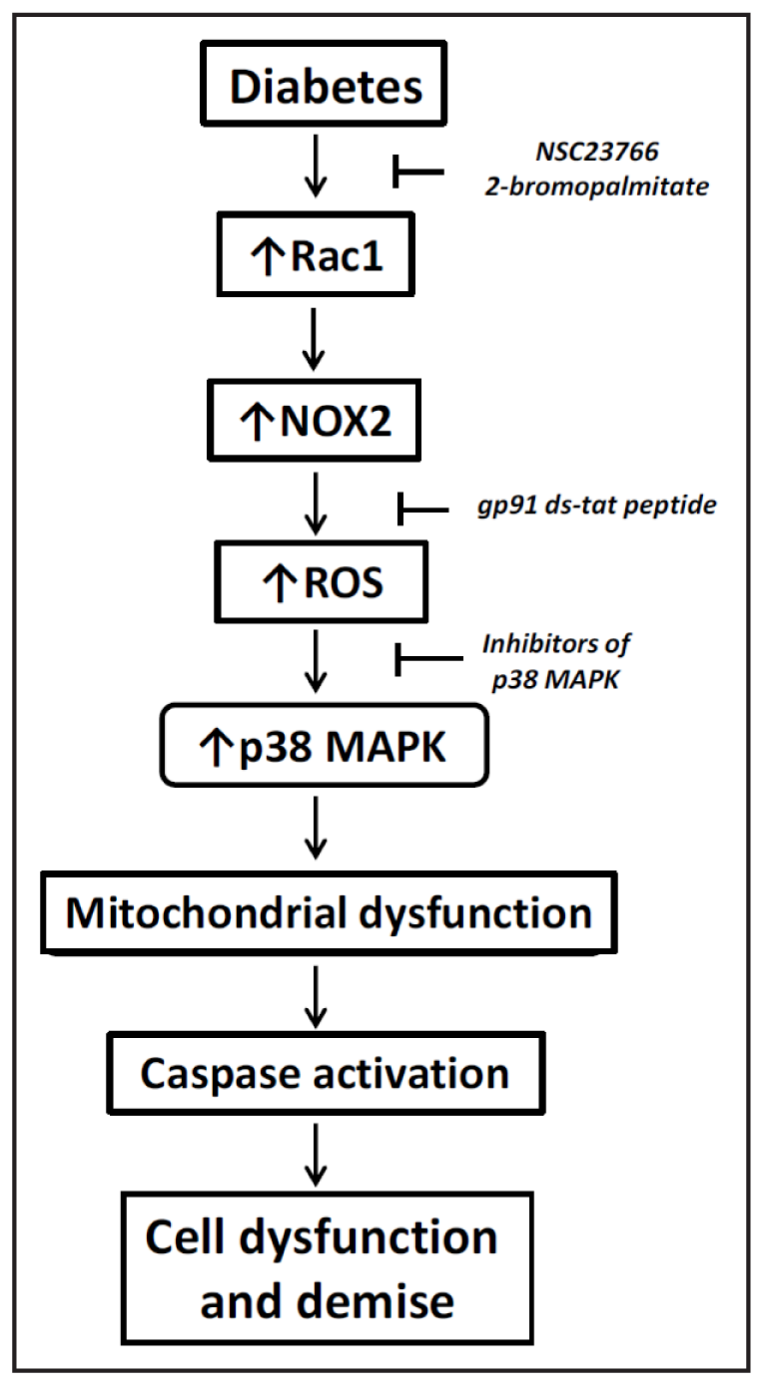
Our proposed model for involvement of Tiam1-Rac1 signaling pathway in the activation of Nox2 and downstream stress kinase, leading to mitochondrial dysfunction and retinal capillary cell apoptosis, and the development of diabetic retinopathy. Based on our recently published evidence [6] and the current findings, we propose a model to highlight the contributory roles of Nox2 in cellular dysfunction induced by hyperglycemia. Chronic exposure of cells to these conditions culminates in the generation of Rac1-Nox2-mediated ROS, which, in turn, leads to activation of stress kinases (p38 MAP kinase) and onset of mitochondrial dysregulation [6]. Alterations in mitochondrial function including leakage of proapoptotic factors (cytochrome-C) into the soluble compartment leads to activation of caspases, which is turn, catalyze the degradation and mislocalization of key structural proteins leading to metabolic dysfunction of the cell. Pharmacological evidence (NSC23766 and gp91 ds-tat peptide; ref.^6^ and current studies) strongly favors the concept that Tiam1-Rac1-Nox2 axis represents one of the areas for the development of novel therapeutics to impede metabolic defects under glucotoxic and diabetic conditions [6]. Our current observations also implicate that protein palmitoyltransferase might represent a potential target for halting glucotoxic effects on retinal endothelial cells and the onset of diabetic retinopathy.

In summary, our current study suggest that hyperglycemic conditions promote activation of Tiam1-Rac1-Nox2 signaling module during early stages of diabetic retinopathy, which leads to activation of stress kinases such as the p38 MAP kinase (Fig. 8). This, in turn, leads to mitochondrial damage resulting in cell apoptosis and the development of diabetic retinopathy. We also propose that palmitoylation of specific signaling proteins (Rac1 and others yet to be identified) represent an important signaling event in this cascade. Thus, strategies to attenuate these signaling steps could offer methods to inhibit the development and progression of retinopathy in the early stages of the disease, thus sparing diabetic patients from losing their sight as a result of this devastating disease.

## Abbreviations

2-BP: 2-bromopalmitate
GAP: GTPase-activating protein
GDI: Guanosine diphosphate dissociation inhibitor
Nox2: NADPH oxidase
NSC23766: [*N*^6^-[2-[[4-(Diethylamino)-1-methylbutyl]amino]-6-methyl-4-pyrimidinyl]-2-methyl-4,6-quinolinediamine trihydrochloride]
p38 MAP kinase: p38 mitogen activated protein kinase
Rac1: Ras-related C3 botulinum toxin substrate 1
ROS: Reactive oxygen species
Tiam1: T-cell lymphoma invasion and metastasis 1
